# Morphogen-defined patterning of *Escherichia coli *enabled by an externally tunable band-pass filter

**DOI:** 10.1186/1754-1611-3-10

**Published:** 2009-07-08

**Authors:** Takayuki Sohka, Richard A Heins, Marc Ostermeier

**Affiliations:** 1 Department of Chemical & Biomolecular Engineering, Johns Hopkins University, 3400 N. Charles St, Baltimore, MD 21212, USA; 2 Present address: Central R&D Laboratories, ASAHI KASEI Corporation, 2-1, Samejima, Fuji, Shizuoka 416-8501, Japan

## Abstract

**Background:**

Gradients of morphogens pattern cell fate – a phenomenon that is especially important during development. A simple model system for studying how morphogens pattern cell behavior would overcome difficulties inherent in the study of natural morphogens *in vivo*. A synthetic biology approach to building such a system is attractive.

**Results:**

Using an externally-tunable band-pass filter paradigm, we engineered *Escherichia coli *cells to function as a model system for the study of how multiple morphogens can pattern cell behavior. We demonstrate how our system exhibits behavior such as morphogen crosstalk and how the cells' growth and fluorescence can be patterned in a number of complex patterns. We extend our cell patterning from 2D cultures on the surface of plates to 3D cultures in soft agarose medium.

**Conclusion:**

Our system offers a convenient, well-defined model system for fundamental studies on how multiple morphogen gradients can affect cell fate and lead to pattern formation. Our design principles could be applied to eukaryotic cells to develop other models systems for studying development or for enabling the patterning of cells for applications such as tissue engineering and biomaterials.

## Background

Morphogens are substances that induce different cell fates in a concentration dependent manner [1,2]. Morphogens are especially important in development because emission of a morphogen from a source can lead to the formation of different cell types in a defined spatial relationship to the source and to each other. The study of natural morphogens during development is challenging owing to the complexity of *in vivo *systems and the extremely low concentrations of most morphogens [3,4]. The engineering of less complex biological systems with position-specific gene expression would provide convenient model systems for testing fundamental concepts and hypotheses of how morphogens can pattern cell fate.

Synthetic biology seeks to create biological systems with desired properties and behaviors [5-7], including the ability to pattern cell behavior. For example, genetic circuits have been built in *E. coli *cells such that fluorescent proteins are expressed only when a particular compound falls within a certain concentration range [8,9]. Such cells function similar to a band-detect filter and the fluorescence pattern that emerges follows the contour lines of the gradient of the compound.

We have recently engineered *E. coli *cells to function as a band-pass filter for β-lactam antibiotics and cellular β-lactamase (BLA) activity in which the position and width of the band can be tuned externally [10]. When challenged to grow in the presence of the β-lactam antibiotic ampicillin (Amp) and tetracycline (Tet), cells grow and fluoresce only if the cellular β-lactamase activity (which is regulated through an IPTG-inducible promoter) falls within a narrow range. In this system, Amp and IPTG function as "morphogens" and can be used together with Tet to pattern cell growth and fluorescence. Amp and IPTG are not true morphogens because *E. coli *is prokaryotic. However, this simple model system can be used for fundamental studies on how morphogens can pattern cell behavior [10]. For example, the opposing effects of Amp and IPTG were used to break the radial symmetry imposed by radial concentration gradients and pattern cell growth in linear and other patterns that lacked radial symmetry (i.e. cells were patterned in defined ways that did not follow contour lines of the concentration gradient).

In this report we describe how the inclusion of an additional promoter that responds to a different cell permeable molecule can result in more complex patterns of growth and gene expression. In response to morphogen/chemical gradients, mixtures of cells with different levels of enzyme activity and different fluorescent protein genes achieved position-specific multi-gene expression patterns resulting in a complex multicolor pattern. In addition, we demonstrate how morphogens in our system can exhibit crosstalk, and we expand our pattering methods from two-dimensions on the surface of a plate to a three-dimensional culture.

## Results and discussion

### Band-pass growth behavior

**Figure 1:**
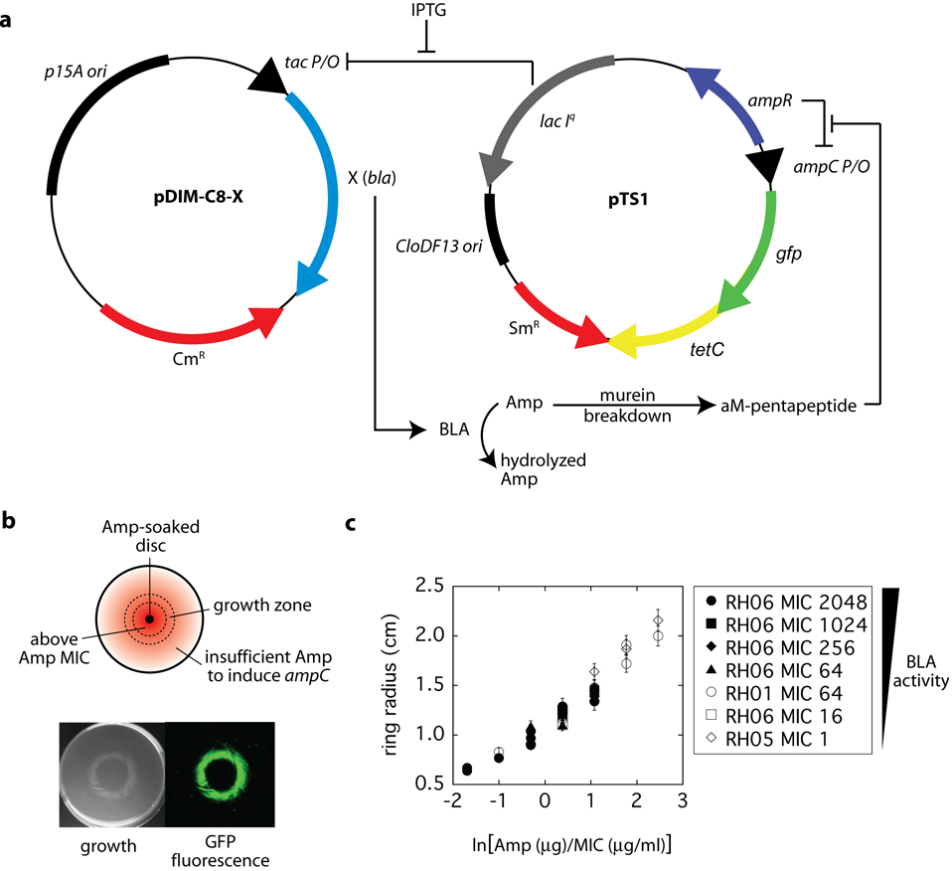
**Principle of the externally tunable bacterial band-pass filter**. **a**, Schematic representation of the plasmid map and gene circuit relationships. BLA expression plasmid (pDIM-C8-X) and reporter plasmid (pTS1) are compatible. In the absence of sufficient cellular β-lactamase (BLA) activity for hydrolysis of ampicillin (Amp), cell wall synthesis is compromised and cells cannot proliferate. In addition, murein breakdown results in the accumulation of aM-pentapeptide. aM-pentapeptide induces the *ampC *promoter via interactions with AmpR [11,12] resulting in the expression of *tetC *(which confers tetracycline resistance) and *gfp *(which encodes the green fluorescent protein). However, the level of Amp necessary to induce *ampC *is lower than the level that prevents the growth of *E. coli *cells. BLA gene expression is regulated through IPTG-induction of the *tac *promoter that is repressed by LacI. **b**, Fluorescence and band-pass growth behaviour on agar plates of *E. coli *cells that have the band-pass filter circuitry. When cells are spread evenly on plates containing Tet/IPTG and an Amp gradient radiating from the center of the plate, a ring of growth and fluorescence appears. **c**, The size of the ring radius (defined as the radius of highest fluorescence) depends on the ratio of the amount of Amp added in the center and the β-lactamase activity of the cells. β-lactamase activity is expressed here as the MIC of Amp in liquid culture [10]. Strains are defined in Additional file 1. The MIC for a strain was set by the concentration of IPTG.

Our engineered *E. coli *cells contain a designed genetic circuit (Fig. 1a) and exhibit band-pass growth behaviour on agar plates containing Tet and a concentration gradient of a β-lactam, such as ampicillin (Amp) [10] (Fig. 1b). High concentrations of Amp (which occur near the center of the plate) prevent cell growth via inhibition of cell wall synthesis. Cells located in regions of low Amp concentration (which occur near the edge of the plate) are growth arrested due to the protein synthesis inhibitory effects of Tet. However, cells located in regions of intermediate Amp concentration can grow due to the Amp-induced expression of the tetracycline resistance gene (*tetC*) conferring resistance to Tet. Amp induces *tetC *through the following mechanism. Transcription of *tetC *and the green fluorescent protein gene (*gfp*) are under the control of the *ampC *promoter, which is suppressed by AmpR. The presence of a beta-lactam such as Amp leads to the accumulation of the cell wall intermediate L1,6-anhydro-N-acetylmuramyl-L-alanyl-D-glutamyl-meso-diaminopimelic acid-D-alanyl-D-alanine (aM-pentapeptide). This compound induces the *ampC *promoter via interactions with AmpR [11,12]. Thus, in the presence of Tet, the cells can grow only in a narrow concentration range of Amp (hence, the system can be described as exhibiting band-pass growth). The level of β-lactamase activity (which is proportionally set by IPTG) determines this narrow concentration range.

Since, intracellular Amp concentration is reduced by cellular β-lactamase activity, the enzyme (BLA) and substrate (Amp) pair acts as an attenuator for the entire system (Fig. 1a). Increased enzyme activity shifts the Amp concentration range required for growth to higher levels. Likewise, an increase in Amp concentration requires an increase in enzyme activity for growth. In this manner, the band-pass filter is externally tunable. In liquid culture, growth requires a particular ratio of β-lactamase activity to Amp[10]. The same is true for growth on agar plates. Ring radius is the same for cells with very different β-lactamase activity provided the ratio of Amp to β-lactamase activity is the same (Fig 1c). Our cells function like a sensor and can be used to detect enzyme activity or substrate concentration in a very narrow range and with wide dynamic range. In addition, both Amp and IPTG function as morphogens [10] that fit Wolpert's French flag model [1]. Thus, the band-pass paradigm transforms *E. coli *into a simple model system for how morphogens affect cell fate during development.

### Crosstalk between different morphogens

**Figure 2:**
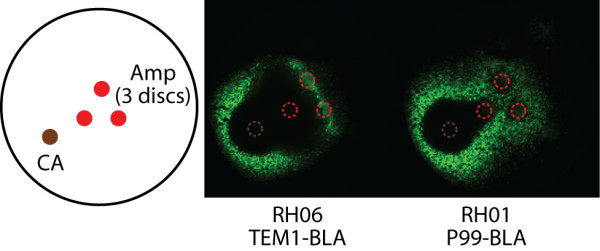
**Morphogen crosstalk**. RH06 cells (expressing TEM1-BLA) or RH01 cells (expressing P99-BLA) were spread evenly on plates containing Tet (15 μg/ml) and IPTG (10 μM or 300 μM, respectively). The level of IPTG was different for the two strains so that their MIC for Amp was the same. Filter paper discs containing Amp or CA were placed at the indicated locations. Both clavulanic acid (CA) and ampicillin (Amp) can induce growth in the presence of Tet. However, CA's differential inhibitory effects of TEM1-BLA and P99-BLA result in different patterns of growth (not shown) and fluorescence.

During development, cells may have to correctly interpret more than one morphogen signal simultaneously. Having two different morphogens makes for a more complex system with potential synergistic or antagonistic effects between the two morphogens. Here, we demonstrate one way in which our prokaryotic model development system can exhibit crosstalk between morphogens. A variety of β-lactams have been shown to induce the *ampC *promoter [12]. We have found that a variety of β-lactams including Amp can induce Tet resistance and pattern cell growth in our system. Although clavulanic acid (CA) has weak antimicrobial activity compared to penicillins, it possesses a β-lactam ring and also could induce Tet resistance and fluorescence in our cells, presumably through the same mechanism as Amp. Thus CA, as it also diffuses well, can act as a morphogen. In addition, CA is a potent inhibitor of TEM1-BLA (a class A β-lactamase) but a weak inhibitor of P99-BLA (a class C β-lactamase) [13-15]. CA inhibits TEM1-BLA irreversibly through the covalent modification of the active site serine residue [13]. Thus, the combination of Amp and CA differentially affected growth patterning of cells expressing TEM1-BLA and P99-BLA owing to CA's different effects on the two β-lactamases (Fig 2).

In this experiment, both strains' ability to hydrolyze Amp was set to be the same by using the IPTG concentration necessary to achieve a minimum inhibitory concentration (MIC) for Amp of 64 μg/ml. In the case of cells expressing P99-BLA, the effect of CA and Amp appears to be largely additive (Fig. 2), as expected. Fluorescence is not seen in between the CA disk and the closest Amp disc because the total concentration of Amp and CA in this region is too high for growth. CA does not affect the concentration of Amp because it is too poor an inhibitor of P99-BLA. In the case of cells expressing TEM1-BLA, crosstalk is observed presumably because CA distorts the Amp gradient through inhibition of TEM1-BLA. This expands the region of no growth beyond what is seen in cells expressing P99-BLA (Fig. 2). CA and Amp are acting synergistically since CA serves to help Amp prevent cell growth by inhibiting TEM1-BLA's Amp degradation activity. These experiments illustrate that our model system can be used to study crosstalk between multiple morphogens. In addition, our system can be used in a simple visual method for characterizing substrate/inhibitor specificity of β-lactamases.

### Patterning of cell growth and fluorescence

**Figure 3:**
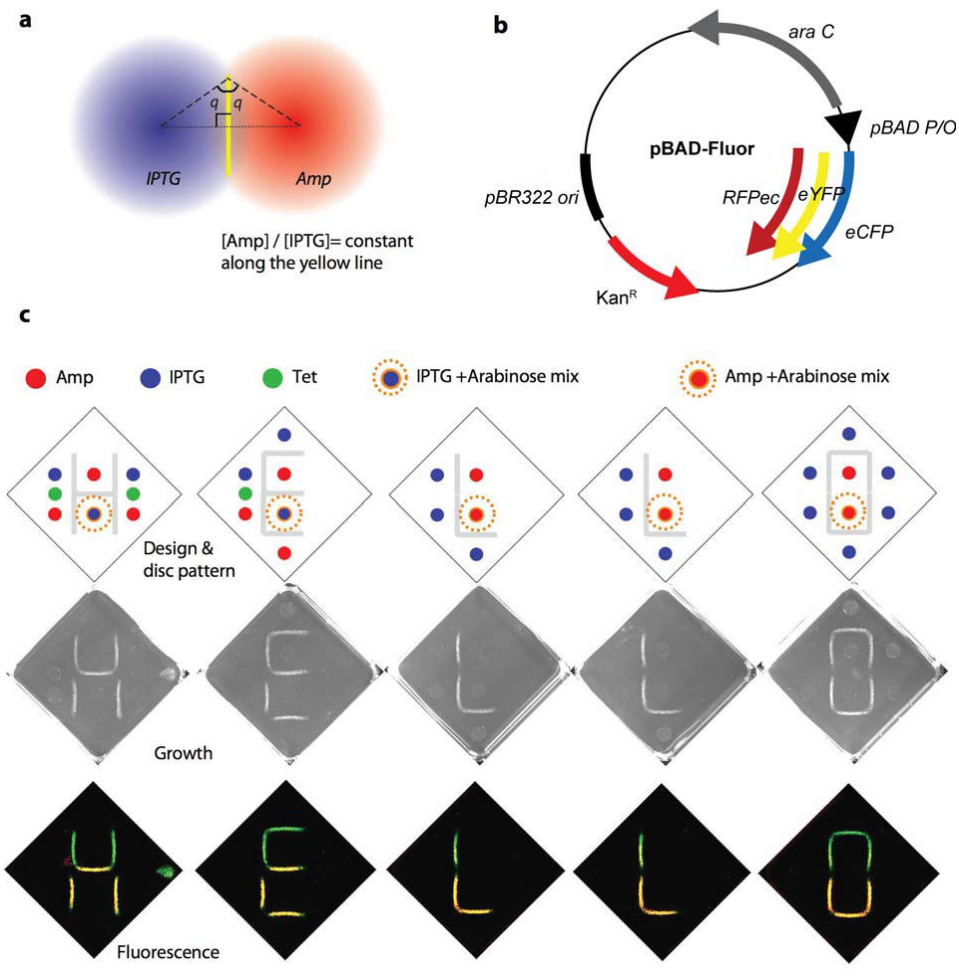
**Patterning cells in multi-colored lines**. **a**, For two overlapping radial gradients, the set of all points equidistant from the center of the two gradients is the location where the ratio of the concentration of the two compounds is a constant. **b**, Schematic of plasmid pBAD-Fluor, the third compatible plasmid harbouring the genes encoding either RFPec, eYFP or eCFP under the arabinose-inducible pBAD promoter. **c**, RH37 cells (harbouring pDIM-C8-P99bla, pTS1 and pBAD-Fluor-RFPec) were spread evenly on plates containing 30 μg/ml Tet. Discs containing Amp, IPTG, Tet and/or arabinose were placed at the indicated positions. Plates were incubated at 37°C. Visible light and merged fluorescent images are shown. The detailed conditions for all plates can be found in Additional file 1.

We have previously demonstrated that overlapping radial gradients of two morphogens with opposing effects (Amp and IPTG) can be used to pattern cells in linear and other patterns that lack radial symmetry [10]. This phenomenon arises because the ratio of BLA activity to Amp concentration that is necessary for growth is constant [10] (Fig. 1c). When two radial concentration profiles overlap, the line consisting of all points equidistance from the two point sources is the set of all locations where the ratio of the concentration of the two compounds is constant (Fig 3a). For point sources of morphogens a set distance apart, there exists an appropriate amount of the morphogen to add at these locations such that the ratio of the two morphogens along the line is at the value required for growth. Note that this analysis assumes equal diffusion constants of the two compounds and neglects consumption of either morphogen. In our system we expect that Amp (but not IPTG) is being consumed. In addition, the cells require a particular ratio of Amp to β-lactamase activity and the relationship between IPTG and β-lactamase activity is not linear. However, if we operate in a regime where the relationship between IPTG and β-lactamase activity is approximately linear the expected linear patterns of growth can be achieved [10] despite the fact that we ignore the degradation of Amp.

Our system can be used to study how simple growth patterns and gene expression profiles can emerge from a homogenous population of cells. However, actual developmental phenomena are much more complicated than this. After cell fate has been patterned by one morphogen, cell behavior might be further regulated by other signals. We sought to capture this in our simple model system by engineering the cells to respond to the signal of another cell permeable molecule. To achieve this, we added a third compatible vector that had a red fluorescent protein gene (*RFPec*) under the control of an arabinose inducible promoter (Fig. 3b). By the appropriate patterning of Amp, IPTG and arabinose, we achieved localized RFPec expression without any change to the growth patterning (Fig. 3c). Thus, in addition to designating where cells grow, we can further pattern the cells with different phenotypes in a location-dependent manner. This is exemplified by our patterning of straight lines of cells that are precisely segmented in color (Fig 3c).

### Multi-cellular separation and patterning of fluorescence

**Figure 4:**
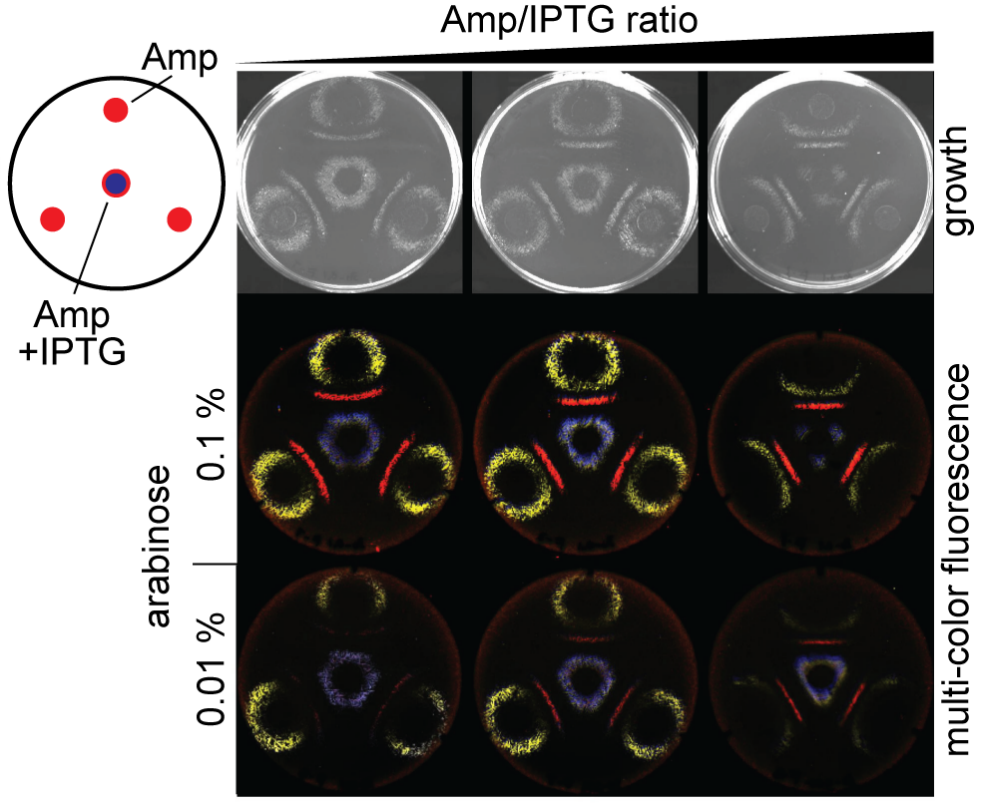
**Multi-cellular separation and patterning of fluorescence**. A 1:1:1 mixture of RH32, RH37, and RH42 cells were spread evenly on plates containing Tet (30 μg/ml) and arabinose (0.1% or 0.01%). Discs of Amp or an Amp/IPTG mix were placed at the indicated locations. Three [Amp]/[IPTG] ratio were tested. Plates were incubated at 37°C. Visible light (of 0.1% arabinose plates) and merged fluorescent images are shown. The rank order of β-lactamase activity of the three strains is RH32 > RH37 > RH42. RH32 fluoresces yellow, RH37 fluoresces red, and RH42 fluoresces blue. The detailed conditions for all plates can be found in Additional file 1.

The band-pass phenotype can be used to separate a mixture of three different cell strains into a rank of order based on their cellular BLA activity and form a bull's-eye pattern [10]. However, cells with the lowest amount of BLA activity, which form the outermost ring, take a long time to grow, presumably due to the time required for Amp to diffuse from the center of the plate. We suspect that the long incubation time results in a broadening of the region of growth and an imperfect separation of the three strains. This is especially true for the outermost ring, since the concentration profile of Amp will be much flatter here than near the Amp source. Through the use of a different pattern of Amp and the inclusion of a gradient of a second morphogen (IPTG) we were able to achieve better separation of the three strains (Fig. 4). We attribute this more defined pattern to the fact that the different cells grow at similar rates in this experiment. In addition, the opposing gradients of Amp and IPTG narrows the region in which the conditions are right for growth (compared to plates in which IPTG is the same concentration everywhere) since a particular ratio of the two compounds is required for growth. To facilitate identification of the three strains, each strain contained a different fluorescent protein gene under the control of an arabinose inducible promoter on a third compatible vector. The cell patterning could be controlled by altering the ratio of the amount of the two morphogens added to the discs. For example, the center ring of growth changed from a hexagon to a triangle as the Amp:IPTG ratio increased. Changing the Amp:IPTG ratio altered the location where the right ratio of Amp to β-lactamase activity is achieved. Decreasing arabinose primarily affect the amount of fluorescent protein produced and not the pattern of growth and fluorescence.

### Three-dimensional growth patterning

**Figure 5:**
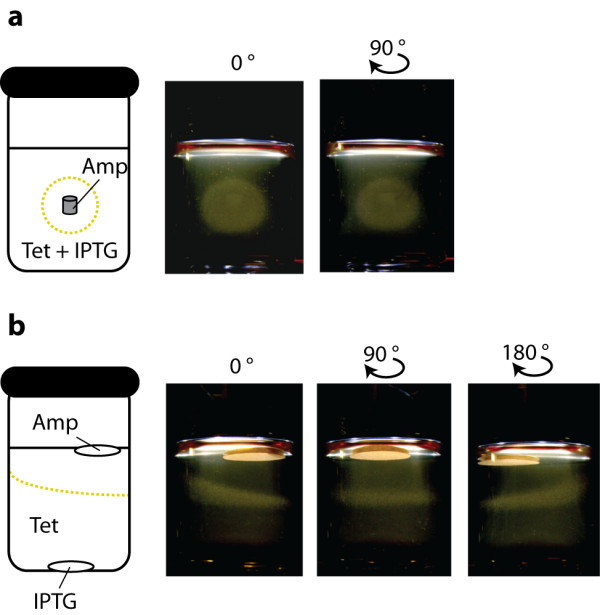
**Three-dimensional growth patterning**. **a**, Growth of bacteria in a sphere. An Amp-impregnated agar gel cylinder was placed in the center of a vial of solidifying soft agarose containing 300 μM IPTG, 30 μg/ml Tet and dilute RH01 cells. The vials were incubated at 37°C overnight and visible images acquired. **b**, Growth of bacteria in a layer. Amp and IPTG disc were placed top and bottom of soft agarose containing 30 μg/ml Tet and dilute RH01 cells. The top disc was placed off center to illustrate that growth depends on the resulting concentration gradients and not the distance from the surface. The vials were incubated at 37°C overnight and visible images acquired. The IPTG disc is not visible in these images.

Plate culture is a simple method for evaluating how morphogens can pattern cell fate. However, a three-dimensional model system would more closely reflect how morphogens affect cells *in vivo *during development. To test our cells in a three-dimensional environment, we suspend a dilute concentration of our *E. coli *cells in soft agarose media in a glass vial and generated morphogen concentration profiles using discs on the surface of the soft agar or small cylinders of high concentration agar embedded in the soft agar. Growth was observed after overnight incubation. When an Amp-impregnated agar cylinder was embedded in the center of Tet-containing soft agar, the expected hollow sphere of growth formed around the cylinder (Fig. 5a). GFP fluorescence could be faintly detected at the top of the sphere and not throughout the entire sphere (data not shown) presumably because GFP's fluorophore requires oxygen for maturation [16] and oxygen is limited in this culturing environment. We also could generate a layer of growth at a defined distance between the top and bottom by placing an IPTG-soaked disc at the bottom and an Amp-soaked top disc on the top of the soft agar (Fig. 5b).

## Conclusion

External tunability and multiple morphogens are important attributes for a useful model developmental system and key for achieving complex patterning of cell behavior. We have demonstrated how a simple band-pass filter genetic circuit in *E. coli *can enable external tunability and can have multiple morphogens with crosstalk and opposing effects. This enables complex patterning including multi-colored line drawing (Fig 3c) and the separation of different cell types into complex patterns based on enzyme activity (Fig 4). Our cells can be patterned in one-dimension (lines), two dimensions (e.g. rings on plates) and three dimensions (e.g. spheres). Our system thus offers a convenient, well-defined model system for fundamental studies on how morphogens gradients can affect cell fate and lead to pattern formation. Our design principles for engineering an externally tunable band-pass filter could be applied to eukaryotic cells to develop other models systems for studying development or for enabling the patterning of cell fate for applications such as tissue engineering and biomaterials.

Such studies would be simplified if morphogen gradients were permanently established instead of evolving over time. In our experiments, the morphogen gradient was continuously changing over the experimental period due to diffusion and degradation. This results in broad, complex gradients that are more difficult to accurately model, and the resulting patterns have relatively large dimensions (i.e. mm to cm scale). With steeper and more stable morphogen gradients, more precise and smaller scale patterning should be possible. The combination of continuous production [8] and rapid degradation of the morphogen at defined locations is one possible strategy for achieving this.

## Methods

### Plasmid

Plasmid constructions for band-pass filter have previously described [10]. Plasmid maps and regulatory relationships are summarized in Fig. 1a. The reporter plasmid (pTS1) has streptomycin (Sm)/spectinomycin (Sp) resistance and carries the CloDF13 replication origin. The β-lactamase expression plasmid pDIM-C8 has chloramphenicol (Cm) resistance and a p15a replication origin, which is compatible with the CloDF13 replicon. All genes cloned into pDIM-C8 are under the control of the IPTG inducible *tac *promoter whose leaky expression was suppressed by LacI from pTS1. Several versions of the pDIM-C8 were constructed and named with an abbreviation for the name of the gene in place of the "X".

pBAD-Fluor (Fig 3b) confers kanamycin (Kan) resistance and has a ColE1 replication origin, which is compatible with the CloDF13 and p15a replicons. The rank of the plasmid copy number is pTS1 > pBAD-Fluor > pDIM-C8 as judged by ethidium bromide staining intensity plasmid DNA isolated from strains harboring all three plasmids. pBAD-Fluor was constructed by replacing the *Amp*^*R *^gene of pBAD/Myc-His A (Invitrogen) with Kan resistant gene with constitutive promoter of pET24d(+) (Novagen) using the BspHI site. The NcoI site (overlaps the ATG start codon) was eliminated and changed to BglII site by PCR based methods. Three version of plasmid pBAD-Fluor were constructed, each of which can express one of three fluorescent proteins (RFPec, eYFP or eCFP). The genes encoding eYFP and eCFP were PCR amplified from commercially available plasmid (Clontech) such that cloning sites BglII and KpnI (for ligation into pBAD-Fluor) were added at the 5' and 3' ends, respectively. The gene encoding RFPec was created by PCR amplification using mutagenic primers and DNA encoding DsRed2 (Clontech) as the template. In addition to introducing the BglII and KpnI sites for cloning, these primers introduced mutations in the 5' region of the *DsRed2 *gene to created *RFPec *– a modified version of *DsRed2 *that expresses very efficiently under the pBAD promoter in *E. coli *[17].

### Strains

Strains and their properties are listed in Additional file 1. *E. coli *SNO301 (*ampD1, ampA1, ampC8, pyrB, recA, rpsL*) was used. The *ampD1 *mutation allows β-lactam hyper-inducible transcription of genes under the control of the *ampC *promoter [12]. The *rpsL *mutation confers streptomycin resistance to this host, the same resistance marker used to maintain pTS1. However, we observed that pTS1 was stably maintained in SNO301 cells in the presence of 100 μg/ml Sm. The *rpsL *mutation does not confer spectinomycin (Sp) resistance. We used Sp instead of Sm in some experiments. Multiple aliquots of frozen cell stocks for plating and growth experiments were prepared from overnight LB cultures at a final optical density at 600 nm of 0.5 and contained 15% glycerol. These stocks were stored at -80°C.

### LB-agar growth experiments

Ten-fold dilutions of frozen stocks (150 μl for 86 mm round plates and 206 μl for 91 mm square plates) were spread evenly over the entire LB-agar (1.5%) plates containing Sm (100 μg/ml), Cm (50 μg/ml) and various concentrations of IPTG, Tet and arabinose. For multi-fluorescent color pattering, plates also contained Kan (30 μg/ml). For the plate in Fig. 4, 50 μl of each of the three strains were mixed before plating. Plate volume was 20 ml for 86 mm round plates and 27.5 ml for 91 mm square plates. Ninety minutes after plating, dry one cm diameter paper discs (six mm discs for Fig. 2) were placed at the desired position using a schematic map laid underneath the plate to ensure reproducible placement. The disks were spotted with Amp, potassium clavulanate (CA), IPTG, arabinose and/or Tet as indicated. Some spots had mixtures of two compounds. The detailed conditions for each experiment can be found in Additional file 1. The plates were incubated at 37°C for 21 hours.

After disc removal growth images were acquired by CCD camera under the white light reflection conditions using ChemiDoc XRS (BioRad). Fluorescence images were acquired by Typhoon 9410 imager (GE Lifescience) using 200 μm resolution and agar top focused (+3 mm). Fluorescent images were taken under the following conditions. For imaging GFP (green): 350 V/488 nm laser excitation (ex), 526 nm short pass (SP) filter emission (em) detection; for imaging eCFP (cyan): 450 V/457 nm ex, 526 nm SP em; for imaging eYFP (yellow): 450 V/532 nm ex, 555 nm band pass filter em; and for imaging RFPec (Red): 500 V/633 nm ex, 580 nm SP em. Greyscale images were converted to color and the contrasts were adjusted by using Image Quant TL software (GE Lifescience). Due to the excitation laser wavelength and emission filter restrictions, it was not possible to completely isolate eCFP/GFP or eYFP/GFP signals. Thus, in Fig. 4, there is a small amount of GFP signal in the eCFP (cyan) and eYFP (yellow) signals. Two colors were merged in Fig. 3c (i.e. GFP (green) and RFPec (Red)). Three colors were merged in Fig. 4 (i.e. eCFP (cyan), eYFP (yellow) and RFPec (Red)).

### Three-dimensional growth patterning

LB medium containing 0.5% (w/v) of low melting point agarose (LMP agarose; Invitrogen) was heated in a microwave until the agarose was molten and was then incubated at 39°C to keep from solidifying. Frozen stocks of RH01 cells were diluted 333-fold into this LB-agarose medium containing Sp (30 μg/ml), Cm (50 μg/ml) and Tet (30 μg/ml). IPTG (300 μM) was included in the sphere growth experiments. For sphere shaped growth (Fig. 5a), the Amp-containing cylinder was embedded in the LB-agarose-cell using a method involving three layers. First, 5 ml of the LB-agarose-cell solution was poured into a glass vial (size: 28 mm × 58 mm; Fisher) and placed on ice until solidified. Immediately after solidification, 0.5 ml of the LB-agarose-cell solution was layered on top. Next, a 2% (w/v) agarose cylinder gel (3 mm diameter × 4 mm height) containing Amp (2.13 mg/ml) was pushed into the center of the unsolidified thin solution. After this layer had solidified and immobilized the cylinder, a final 5 ml of the LB-agarose-cell solution was layered on top. After the final layer had solidified, the vial was incubated for 21 hr at 37°C. For layered growth (Fig. 5b), first 600 μl of 2% agarose was placed in the bottom of the vial. After solidification, a paper disc (1 cm) was placed on the center and 15 μl of 300 mM IPTG was spotted on it. The vial was incubated at room temperature for 6 hrs. Next, 10 ml of LB-agarose-cell solution was layered on top and the vial was placed on ice until the layer solidified. A second paper disc was placed on the gel, to which 15 μl of 12.5 mg/ml Amp was spotted. The vial was incubated for 21 hr at 37°C. In both experiments, the heights of the gel were about 2 cm. Growth images were acquired by digital camera (Canon) equipped with a macro lens under the white LED light from the bottom reflection conditions using super-macro mode. Contrasts and color balances were adjusted by using Microsoft photo editor to better visualize the growth pattern.

Strain descriptions and detailed disc pattern information can be found in Additional file 1.

## Competing interests

The authors declare that they have no competing interests.

## Authors' contributions

TS, RAH and MO designed experiments. TS performed experiments. TS and MO wrote the manuscript. All authors read and approved the final manuscript.

## Supplementary Material

Additional file 1 ***E. coli *strains and templates for growth patterns**. The table provides a description of all strains used. The figure provides a template for all growth patterns. Click here for file
