# Supplementary material for: Morphogen-defined patterning of Escherichia coli enabled by an externally tunable band-pass filter
Source: J Biol Eng. 2009 Jul 8;3:10. doi: 10.1186/1754-1611-3-10 (PMC2715369; doi:10.1186/1754-1611-3-10)
Supplement: Additional file 1 — E. coli strains and templates for growth patterns. The table provides a description of all strains used. The figure provides a template for all growth patterns. [file 1754-1611-3-10-S1.pdf]

Morphogen-defined patterning of *Escherichia coli* enabled by an externally tunable band-pass filter

Supplementary Information

Takayuki Sohka, Richard A. Heins and Marc Ostermeier

Table S1. *E. coli* strains

Strain [a]	Plasmid 1[b]	Plasmid 2[b]	Plasmid 3[c]	Ref.
RH01	pTS1	pDIMC8-P99bla	-	[1]
RH05	pTS1	pDIMC8-MBP	-	[1]
RH06	pTS1	pDIMC8-TEM1bla	-	[1]
RH32	pTS1	pDIMC8-TEM1bla	pBAD-Fluor-eYFP	this work
RH37	pTS1	pDIMC8-P99bla	pBAD-Fluor-RFPec	this work
RH42	pTS1	pDIMC8-MBP	pBAD-Fluor-eCFP	this work

[a] All strains are derived from SNO301(*ampD1, ampA1, ampC8, pyrB, recA, rpsL*)
[b] Plasmids described in Figure 1a and reference [1].
[c] Plasmids described in Figure 3b.

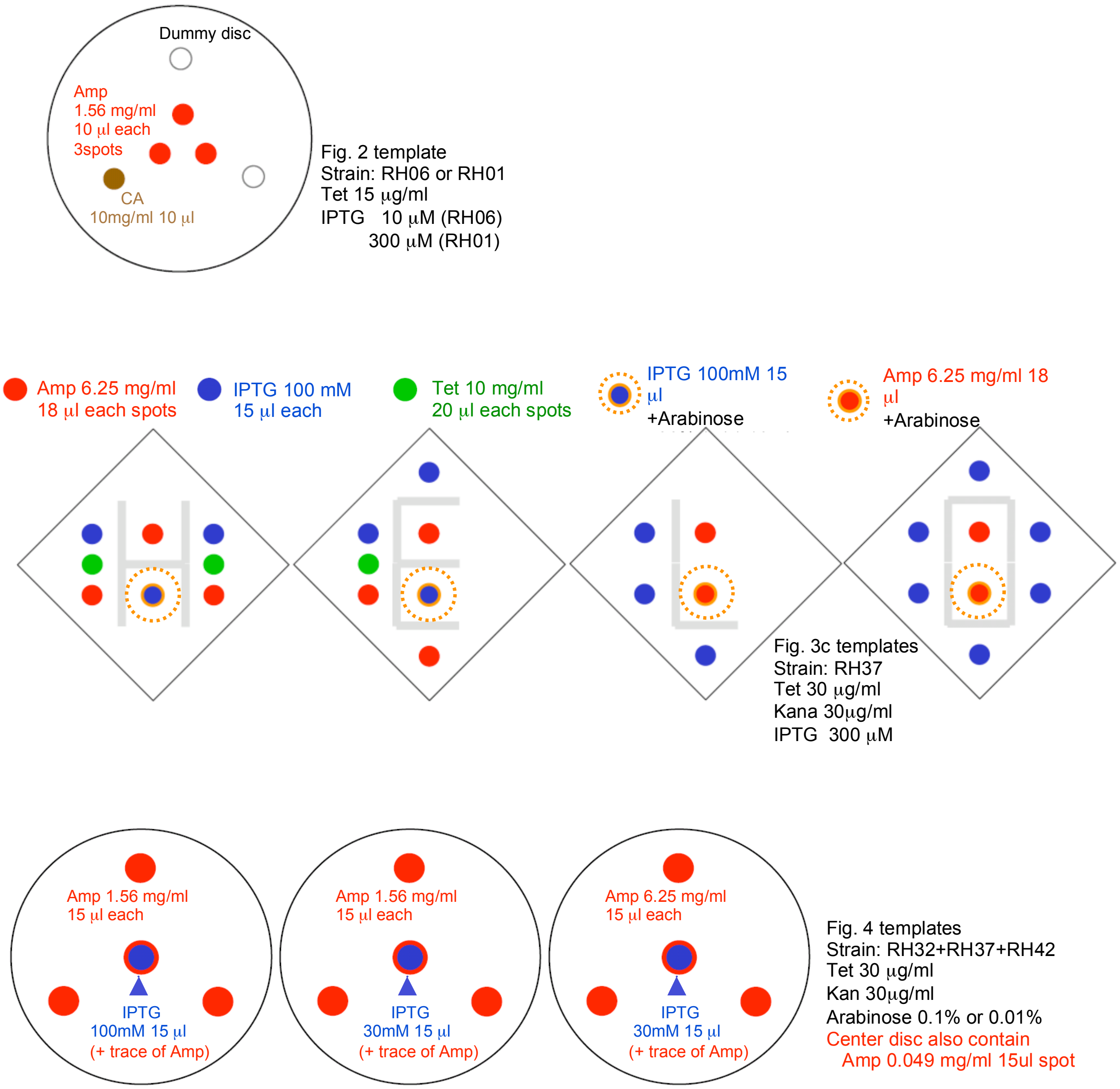

Figure S1. Templates for plate growth patterns. All plates contained 100 µg/ml Sm and 50 µg/ml Cm.

References

1. Sohka T, Heins RA, Phelan RM, Greisler JM, Townsend CA, Ostermeier M; **An externally-tunable bacterial band-pass filter for enzymatic activity.** *Proc Natl Acad Sci USA* 2009, **106**:10135-10140.
